# Preoperative Main Pulmonary Artery Diameter Indexed to Body Surface Area Independently Predicts Mortality After Transcatheter Aortic Valve Implantation in a Chinese Population

**DOI:** 10.31083/RCM50699

**Published:** 2026-06-25

**Authors:** Jiaqi Zhang, Chengwei Chi, Huan Qin, Simiao Tian, Jiyi Liu, Shulong Zhang, Jihong Liu, Ruiqin Zhang, Enze Jin

**Affiliations:** ^1^Cardiovascular Medical Department, Harbin Medical University, 150000 Harbin, Heilongjiang, China; ^2^Cardiovascular Medical Department, The Fourth Affiliated Hospital of Harbin Medical University, 150000 Harbin, Heilongjiang, China; ^3^Department of Cardiology, Affiliated Zhongshan Hospital of Dalian University, 116000 Dalian, Liaoning, China; ^4^Department of Medical Record and Statistics, Affiliated Zhongshan Hospital of Dalian University, 116000 Dalian, Liaoning, China

**Keywords:** aortic valve stenosis, transcatheter aortic valve implantation, tomography, X-ray computed, pulmonary artery, pulmonary hypertension

## Abstract

**Background:**

Pulmonary hypertension (PH) commonly accompanies severe aortic stenosis and adversely affects outcomes after transcatheter aortic valve implantation (TAVI). However, the prognostic value of computed tomography-derived pulmonary artery remodeling, particularly the main pulmonary artery diameter (mPAD) indexed to body surface area (mPAD/BSA), remains uncertain. Therefore, this study aimed to investigate the prognostic significance of mPAD/BSA in patients with severe aortic stenosis and concomitant PH undergoing TAVI.

**Methods:**

We retrospectively analyzed 122 consecutive patients undergoing TAVI, while the subgroup of 103 patients referred to those with pulmonary hypertension. Pulmonary artery systolic pressure (PASP) was estimated by transthoracic echocardiography, while mPAD was quantified on multidetector computed tomography (MDCT) and indexed to BSA. Patients were stratified according to postoperative changes in PASP. Echocardiographic and imaging parameters were compared across PH severity categories.

**Results:**

Postoperative PASP improvement was observed in 71 patients (68.9%). Patients with PASP improvement had smaller baseline mPAD and mPAD/BSA but higher baseline PASP than those who showed no improvement (all *p* < 0.05). Although PASP improved in patients with pre-existing PH, the extent and trajectory of recovery varied according to baseline PH severity. In contrast, left ventricular hemodynamics improved uniformly across all groups, whereas right heart remodeling and pulmonary artery structural changes remained limited. In the multivariable logistic regression analysis, baseline atrial fibrillation (AF) (*p* = 0.009), preoperative first-degree atrioventricular block (AVB) (*p* = 0.032), and preoperative ≥ moderate tricuspid regurgitation (TR) (*p* = 0.037) were independently associated with a lower risk of postoperative PASP non-improvement. In contrast, preoperative ≥ moderate aortic regurgitation was associated with a higher risk of PASP non-improvement (*p* = 0.022). Notably, preoperative MDCT-derived mPAD/BSA independently predicted 1-year all-cause mortality (HR 1.29, 95% CI 1.01–1.64; *p* = 0.041), providing incremental prognostic information alongside echocardiographic PASP.

**Conclusions:**

PH secondary to aortic stenosis is only partially reversible after TAVI, with greater improvement in mild-to-moderate disease. Preoperative mPAD/BSA is a novel MDCT-based structural biomarker that independently predicts 1-year all-cause mortality beyond PASP alone. Integrating pulmonary vascular imaging with echocardiography may improve risk stratification and inform procedural timing in patients undergoing TAVI.

## 1. Introduction

Pulmonary hypertension (PH) is a common comorbidity in patients with severe aortic stenosis (AS) and is associated with adverse clinical outcomes following transcatheter aortic valve replacement [1]. Transcatheter aortic valve implantation (TAVI) has been established as a treatment strategy for individuals with symptomatic, advanced AS who are deemed unsuitable for, or at elevated procedural risk from, conventional surgical aortic valve replacement (SAVR) [2,3,4]. Selection of candidates for TAVI is guided by both clinical characteristics and anatomical considerations, with formal risk stratification incorporated as a central element of procedural planning [5,6]. Noninvasive assessment of PH based on transthoracic echocardiography (TTE) and computed tomographic angiography (CTA) shows strong concordance with pulmonary artery systolic pressure (PASP) values obtained through right heart catheterization across diverse cardiovascular conditions [7,8]. Although relief of left ventricular (LV) outflow obstruction may reduce pulmonary artery pressure, the extent of PH reversibility differs considerably among individuals, and persistent PH after TAVI continues to be a key determinant of prognosis. Therefore, precise evaluation of pulmonary vascular involvement is critical for risk stratification and the optimization of procedural timing in this population.

In severe AS, PH is typically regarded as a marker of disease decompensation, reflecting the failure of LV adaptive capacity and progression to a heart failure phenotype driven by valve-related myocardial stress or injury [9]. Nevertheless, PH may also be observed in a subset of patients in whom its presence is not attributable to the valvular pathology. As AS advances, impairment of left atrial (LA) function and the development of functional mitral regurgitation may be observed, leading to sustained increases in LA pressure and mean pulmonary artery wedge pressure (mPAWP) [10]. Prolonged exposure to markedly elevated wedge pressures can subsequently induce structural remodeling of the pulmonary vascular bed.

Previous investigations of pulmonary circulatory status in individuals undergoing TAVI have predominantly relied on invasive right heart catheterization data to assess PH severity and track hemodynamic response after TAVI [11]. However, PASP mainly reflects the acute pressure load and may not fully capture intrinsic pulmonary vascular remodeling, especially in advanced disease. Structural alterations of the pulmonary arteries reflect cumulative vascular injury and may offer complementary prognostic information beyond short-term pressure variation. Multidetector computed tomography (MDCT) enables quantitative assessment of pulmonary artery morphology, including main pulmonary artery diameter (mPAD) and its indexation to body surface area (mPAD/BSA). However, the clinical significance of these imaging biomarkers in patients treated with TAVI remains incompletely defined.

Prior studies have documented links between pulmonary artery enlargement and PH severity. Direct examination of MDCT-derived pulmonary artery remodeling in the TAVI setting remains sparse, however, and the incremental prognostic contribution of mPAD/BSA beyond echocardiographic PASP remains to be clarified. In addition, the determinants of non-improvement in postoperative PASP and their associations with pulmonary vascular architecture and right-sided cardiac remodeling require further investigation.

Accordingly, the present study was designed to systematically evaluate pulmonary hemodynamic and structural alterations after TAVI across varying PH severities using integrated echocardiographic and MDCT-based assessment. Additional aims were to identify clinical factors associated with lack of improvement in postoperative PASP, and to determine whether preoperative mPAD/BSA provides independent prognostic information for 1-year mortality beyond PASP alone. We hypothesized that pulmonary artery structural remodeling assessed by MDCT constitutes a distinct and clinically meaningful marker of disease burden that may improve risk stratification in patients treated with TAVI.

## 2. Subjects and Methods

### 2.1 Study Population

#### 2.1.1 Patients Selection

For this study, we recruited patients diagnosed with PH with severe AS and implanted with Venus A Valve (Venus MedTech, Hangzhou, China), VitaFlow valve (Microport MedTech, Shanghai, China) or TaurusElite valve (PEIJIA MedTech, Shanghai, China) at the Heart Center of Affiliated Zhongshan Hospital, Dalian University, from January 2019 to January 2025. The data for this research was accessed in November 2025. Indications for TAVI in selected patients were carefully evaluated by a multidisciplinary heart team, including specialists from cardiology, cardiac surgery, echocardiography, radiology, interventional cardiology, anesthesiology, and cardiopulmonary bypass [12]. All echocardiographic examinations were conducted by two experienced echocardiologists.

#### 2.1.2 Inclusion Criteria

Patients were eligible for inclusion if they met the following criteria: (1) a diagnosis of severe AS according to the European Society of Cardiology/European Association for Cardio-Thoracic Surgery guidelines, defined by an aortic valve area (AVA) ≤1.0 cm^2^, indexed AVA ≤0.6 cm^2^/m^2^, or peak aortic jet velocity (Vmax) ≥4.0 m/s; (2) intermediate- or high-risk surgical candidates, or those with a Society of Thoracic Surgeons (STS) score >4; and (3) the presence of symptoms attributable to severe aortic stenosis. All patients attended a 1-year follow-up and received TTE and MDCT [13,14].

#### 2.1.3 Exclusion Criteria

Patients were excluded if they had any of the following conditions: (1) active infective endocarditis, acute aortic dissection, recent myocardial infarction, left ventricular thrombus, or hypertrophic cardiomyopathy; and (2) patients with an estimated life expectancy of <1 year [15]. Patients with inadequate echocardiographic image quality (n = 1) during follow-up and those who were followed at another referral center (n = 2) were excluded. In addition, MDCT parameters were not obtained in a timely manner in two patients after TAVR during the six-month follow-up, as illustrated in **Supplementary Fig. 1**. The aortic valve phenotype was categorized according to cusp number, raphe count, and raphe orientation [16]. Aortic valve morphology and annular measurements were assessed using echocardiography and MDCT.

#### 2.1.4 Study Population

Accordingly, 103 participants met the study eligibility criteria and were assigned to two groups based on changes in PASP at discharge. PASP was measured within 24 hours prior to hospital discharge. Improvement in PH was defined as the recovery of PASP before hospital discharge. Patients who achieved normalization or a clinically meaningful reduction of PASP at discharge were classified into the PH improvement group, whereas all remaining patients, including those with persistently elevated or worsened PASP, were assigned to the non-improvement group. This study received approval from the Ethics Committee of the Affiliated Zhongshan Hospital of Dalian University (KY2025-172-1). No industry funding was involved. All procedures were conducted in accordance with the principles outlined in the Declaration of Helsinki.

### 2.2 Patient Classification and Data Collection

According to the recommendations of the ESC/ERS PH guidelines for echocardiographic assessment, PASP severity was categorized as mild (35–49 mmHg), moderate (50–69 mmHg), or severe (≥70 mmHg). Patients were subsequently classified into two groups (PASP improvement group: 71 patients; Non-PASP improvement: 32 patients) based on changes in PASP severity at discharge. PASP improvement was defined as a reduction in the category of severity, specifically transitions from severe PH to mild, moderate, or normal PASP. Patients with unchanged or worsened PASP severity were categorized as having no PASP improvement.

Clinical data were retrospectively retrieved from electronic medical records and the catheterization laboratory database. Baseline demographic and clinical characteristics were recorded before intervention. Patients underwent TTE and MDCT both before and after TAVI.

### 2.3 Treatment and Follow-Up

#### 2.3.1 TTE and MDCT

TTE examinations were conducted by experienced cardiologists in a dedicated echocardiography laboratory. All studies were acquired using a Philips EPIQ 7 platform with a 2.5-MHz transducer by a single echocardiographer. Digitally archived datasets were independently evaluated by two echocardiologists. The readers were fully blinded to patient clinical information. PASP was estimated using continuous-wave Doppler–derived peak tricuspid regurgitation velocity (TRV) and calculated according to the modified Bernoulli equation (PASP = 4 × TRV^2^ + right atrial pressure) [17]. Right atrial pressure was estimated based on the inferior vena cava diameter and inspiratory collapse [18]. All echocardiographic measurements were performed in accordance with current guidelines for right heart assessment [19].

MDCT was performed using a 128-slice scanner. Image acquisition was conducted with a slice thickness of 0.5–0.625 mm using retrospective electrocardiogram (ECG) gating. Contrast-enhanced imaging was performed following intravenous administration of contrast medium, with scan timing determined using a bolus-tracking technique. The region of interest was placed in the pulmonary artery or aorta, and image acquisition was automatically triggered at a predefined threshold (e.g., 100 HU). Main pulmonary artery diameter was measured on MDCT images at the level immediately proximal to the pulmonary artery bifurcation [20]. Multiplanar reconstruction was applied to generate a cross-sectional plane perpendicular to the long axis of the main pulmonary artery to avoid oblique measurements. The maximal short-axis inner-to-inner diameter was recorded for analysis [21]. All measurements were independently performed by two blinded observers, and discrepancies were resolved by consensus through joint review to ensure measurement accuracy.

#### 2.3.2 TAVI Procedure

The transfemoral approach was preferred for TAVI. However, alternative access routes were selected when transfemoral access was not feasible due to small vessel caliber, marked tortuosity, or severe calcification. Access was individualized as transfemoral (TF), transapical (TA), or transaxillary (TAX) based on femoral and iliac anatomy, or a history of peripheral artery disease (PAD) [22]. All patients underwent TAVI under general anesthesia. Prosthesis type was selected according to device availability at the time of intervention. Valve sizing was determined by annular measurements and landing-zone calcification. Balloon pre- or post-dilation was performed at operator discretion. In most cases, aortic root angiography was obtained before balloon inflation. Pre-dilation was routinely performed except in cases of extensive calcification. An additional valve was implanted in the presence of moderate or severe aortic regurgitation after balloon inflation.

#### 2.3.3 Study Endpoints and Follow-Up Time

TTE and MDCT were assessed at three predefined time points: preoperatively (baseline), postoperatively within 24 hours prior to hospital discharge (before discharge), and at 6-month follow-up. Postoperative PASP was defined as the measurement obtained within 24 hours before discharge. PASP improvement was defined as a reduction in PASP severity category within 24 hours prior to hospital discharge after TAVI compared with baseline. The perioperative endpoint events were also documented. Clinical outcomes were adjudicated according to the Valve Academic Research Consortium-3 (VARC-3) criteria [23]. The primary endpoints comprised all-cause and cardiovascular mortality.

### 2.4 Statistical Analysis

Continuous variables were assessed for normality before analysis. Normally distributed variables are presented as mean ± standard deviation, whereas non-normally distributed variables are expressed as median with interquartile range (IQR). Because most continuous variables in the present study were approximately normally distributed, the majority of measurement data are reported as mean ± standard deviation. Categorical variables are reported as counts and percentages. Missing data were minimal (<5%). Therefore, a complete-case analysis was performed, and cases with missing values were excluded. Given the low proportion of missing data, this approach is unlikely to have introduced significant bias. Categorical variables were compared using the χ^2^ test or Fisher’s exact test, as appropriate. Group differences were assessed using paired Student’s *t* test (pre–post comparisons) or unpaired Student’s *t* test (independent groups) for normally distributed variables and the Mann–Whitney *U* test or Wilcoxon signed-rank test for nonparametric data.

Group allocation (No PH, Mild-to moderate PH, Severe PH) was the between-group factor. Time was the within- group factor (baseline, discharge, 6-month follow-up). Compared with the same group at baseline, ^a^*p* < 0.05, compared with the same group at discharge after TAVR, ^b^*p* < 0.05, values in bold are statistically significant. Compared with the No PH group at the same follow-up time, ^A^*p* < 0.05, compared with the Mild-to-moderate PH group at the same follow-up time, ^B^*p* < 0.05, *p* values in bold are statistically significant. If the sphericity assumption was violated, the conservative Greenhouse–Geisser correction was applied. Global effects were tested, and post hoc pairwise comparisons were conducted using Bonferroni correction to adjust for multiple comparisons.

The independent variables included in the multivariable logistic regression analysis were selected based on clinical relevance, prior literature, and statistical significance in univariable analysis (*p* < 0.10). Given the limited sample size and number of events, the number of variables included in the model was restricted to minimize the risk of overfitting. Postoperative PASP improvement was analyzed as a binary outcome. Univariable and multivariable logistic regression analyses were performed to identify factors associated with lack of PASP improvement. Time-to-event outcomes were assessed using Kaplan–Meier survival curves and compared by the log-rank test. Cox proportional hazards regression was used to determine independent predictors of 1-year all-cause and cardiac mortality. To reduce the risk of overfitting, the number of covariates included in the multivariable Cox model was restricted to clinically relevant variables. In addition, Firth’s penalized likelihood method was applied to mitigate small-sample bias. The proportional hazards assumption was assessed using Schoenfeld residuals. Model discrimination was evaluated using the C-statistic, and the incremental prognostic value of mPAD/BSA beyond PASP was further assessed. Figures were generated using GraphPad Prism version 8.0 (GraphPad Software, San Diego, CA, USA). SPSS Statistics version 25.0 (IBM Corp., Armonk, NY, USA) was used for data analysis, and a two-sided *p*-value < 0.05 was considered statistically significant.

## 3. Results

### 3.1 Patient Baseline Characteristics in the Groups of Patients With or Without PASP Improvements

**Fig. 1.**
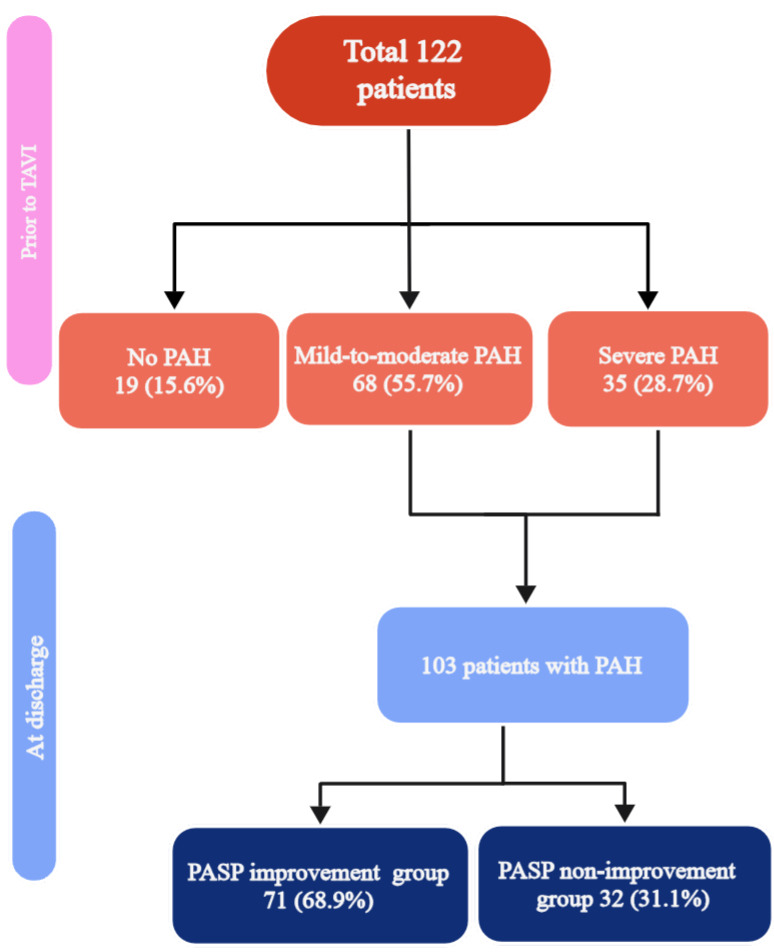
**Study flow chart**. PH, pulmonary hypertension; PASP, pulmonary artery systolic pressure.

In total, as shown in Table 1 and Fig. 1, 103 patients who had detailed clinical information and imaging data of baseline characteristics (mean age 75.0 ± 7.2 years, 68.9% patients with PASP improvements) were enrolled in our study. All the patients underwent TAVI procedures; 71 patients had PASP improvements, and 32 patients did not have PASP improvements before hospital discharge.

**Table 1.** **Baseline characteristics**.

		PASP improvement		*p* value
		Yes (n = 71)	No (n = 32)	
Age (years)		75.0 ± 7.2	76.3 ± 8.1	0.423
BAV		26 (36.6)	14 (43.8)	0.562
Male		34 (47.9)	16 (50.0)	0.843
BSA (m^2^)		1.78 ± 0.19	1.80 ± 0.18	0.625
NYHA class Ⅲ/Ⅳ		55 (77.5)	28 (87.5)	0.233
CrCl, mL/minute		78.28 ± 32.86	68.04 ± 34.38	0.196
Comorbidities:				
	Dyslipidemia	36 (50.7)	22 (68.8)	0.087
	Diabetes	26 (36.6)	13 (40.6)	0.698
	Hypertension	49 (69.0)	19 (59.4)	0.339
	COPD	1 (1.4)	4 (12.5)	0.054
	Stroke	31 (43.7)	12 (37.5)	0.557
	PVD	18 (25.4)	9 (28.1)	0.767
	CAD	43 (60.6)	18 (56.3)	0.680
	Atrial fibrillation	16 (22.5)	15 (46.9)	**0.013**
	Previous valvular replacement surgery	1 (1.4)	2 (6.3)	0.252
	Prior CABG	1 (1.4)	1 (3.1)	0.527
	Need for urgent aortic valvular intervention	1 (1.4)	1 (3.1)	0.527
Risk evaluation:				
	EuroScore Ⅱ	7.06 ± 7.07	7.60 ± 6.56	0.710
	STS Mortality	4.71 ± 3.96	5.44 ± 4.51	0.405
Electrocardiogram:				
	Sinus	60 (84.5)	22 (68.8)	0.066
	Other atrial rhythm	2 (2.8)	1 (3.1)	1.000
	1° AVB	8 (11.3)	9 (28.1)	**0.033**
	LBBB	4 (5.6)	0	0.413
	RBBB	10 (14.1)	3 (9.4)	0.730
	LAFB	4 (5.6)	1 (3.1)	0.958
Echocardiogram:				
	PASP (mmHg)	63.31 ± 16.44	56.50 ± 14.08	**0.035**
	AR ≥ moderate	46 (64.8)	14 (43.8)	**0.045**
	MR ≥ moderate	38 (53.5)	17 (53.1)	0.970
	TR ≥ moderate	24 (33.8)	19 (59.4)	**0.015**
	MPG (mmHg)	47.69 ± 18.59	53.47 ± 31.14	0.335
	AVA (cm^2^)	0.70 ± 0.22	0.69 ± 0.20	0.819
	AVA/BSA (cm^2^/m^2^)	0.39 ± 0.13	0.38 ± 0.11	0.663
	LVEF (%)	51.93 ± 12.47	51.13 ± 13.24	0.767
	LVEDD (mm)	51.31 ± 7.05	52.59 ± 10.37	0.527
	RAD (mm)	46.90 ± 9.19	47.13 ± 8.50	0.907
	RVD (mm)	48.89 ± 12.05	47.34 ± 9.90	0.527
	AAD (mm)	20.38 ± 2.62	20.53 ± 2.36	0.781
	LVPWT (mm)	12.10 ± 1.98	12.13 ± 2.25	0.952
	IVSD (mm)	13.27 ± 2.56	13.19 ± 2.35	0.880
	LVMi (g/m^2^)	149.58 ± 38.86	155.02 ± 46.60	0.539
	RWT	0.51 ± 0.12	0.51 ± 0.14	0.950
MDCT:				
	mPAD (mm)	25.73 ± 4.25	28.00 ± 3.14	**0.008**
	mPAD/BSA (mm/m^2^)	14.59 ± 2.70	15.75 ± 2.62	**0.045**

BAV, bicuspid aortic valve; BSA, body surface area; NYHA, New York Heart Association; CrCl, creatinine clearance; COPD, chronic obstructive pulmonary disease; PVD, peripheral vascular disease; CAD, coronary artery disease; CABG, coronary artery bypass graft; STS, Society of Thoracic Surgeons; 1° AVB, first-degree atrioventricular block; LBBB, left bundle branch block; RBBB, right bundle branch block; LAFB, left anterior fascicular block; PASP, pulmonary artery systolic pressure; AR, aortic regurgitation; MR, mitral regurgitation; TR, tricuspid regurgitation; MPG, mean aortic gradient; AVA, aortic valve area; LVEF, left ventricular ejection fraction; LVEDD, left ventricular end-diastolic dimension; RAD, right atrial diameter; RVD, right ventricular diameter; AAD, aortic valve annulus diameter; LVPWT, left ventricular posterior wall thickness; IVSD, interventricular septum in diastole; LVMi, left ventricular mass index; RWT, relative wall thickness; mPAD, main pulmonary artery diameter; *p-*values in bold are statistically significant.

Patients with PASP improvements exhibited a higher prevalence of atrial fibrillation and concomitant moderate or greater aortic and tricuspid regurgitation, which reached statistical significance (*p* < 0.05). First-degree atrioventricular block (1° AVB) was more frequently observed in the non-improvement group than in the PH improvement group (*p* < 0.05). At baseline, PASP was significantly higher in the PH improvement group than in the non-improvement group (63.31 ± 16.44 vs. 56.50 ± 14.08 mmHg, *p* = 0.035). Patients in the PH improvement group had significantly smaller main pulmonary artery diameter (25.73 ± 4.25 vs. 28.00 ± 3.14 mm, *p* = 0.008) and pulmonary artery diameter indexed to body surface area (14.59 ± 2.70 vs. 15.75 ± 2.62 mm/m^2^, *p* = 0.045) compared with those in the non-improvement group.

### 3.2 Procedural Characteristics and Outcomes

As shown in Table 2, compared with those of patients with PH improvements, the mild paravalvular leak and permanent pacemaker implantation in patients with PH non-improvements were comparable in Table 2. Moderate or severe paravalvular leak occurred less frequently in the PH improvement group than in the non-improvement group (2.8% vs. 15.6%, *p* = 0.049).

**Table 2.** **Procedural data**.

	PASP improvement		*p* value
	Yes (n = 71)	No (n = 32)	
Mild paravalvular leak	2 (2.8)	3 (9.4)	0.348
Moderate/severe paravalvular leak	2 (2.8)	5 (15.6)	**0.049**
PPM	9 (12.7)	8 (25.0)	0.119

PPM, permanent pacemaker implantation; *p*-values in bold are statistically significant.

### 3.3 Comparative Analysis of TTE and MDCT Pre- and Post-TAVI in Patients With or Without PH

#### 3.3.1 Right Ventricular Hemodynamic Parameters

In the repeated-measures analysis, significant effects of group, time, and group*time interaction were observed for PASP (all *p* < 0.001), indicating that both baseline PH severity and follow-up time significantly influenced PASP trajectories after TAVI, as shown in Table 3.

**Table 3.** **Comparison of echocardiography parameters in patients with PH and without PH at baseline, at discharge and at six-month follow-up**.

		No PH (n = 19)	Mild-to-moderate PH (n = 68)	Severe PH (n=35)	F value	*p *value
PASP (mmHg, *x̅* ± *s*)						
	Baseline	26.84 ± 3.29	51.76 ± 9.21^A^	79.51 ± 8.78^AB^	257.073	**<0.001**
	Discharge	27.68 ± 2.95	38.91 ± 11.92^aA^	44.49 ± 17.95^aA^	10.039	**<0.001**
	6 M follow-up	28.68 ± 4.53	35.28 ± 10.99^ab^	38.63 ± 16.98^abA^	3.970	**0.021**
F value		0.169	51.345	173.938		
*p *value		0.844	**<0.001**	**<0.001**		
Global text						
	group (F value, *p *value)	56.390, **<0.001**				
	time (F value, *p *value)	115.263, **<0.001**				
	group*time (F value, *p *value)	45.610, **<0.001**				
MPG (mmHg, *x̅* ± *s*)						
	Baseline	53.00 ± 19.22	52.26 ± 24.71	44.09 ± 19.18	1.727	0.182
	Discharge	13.11 ± 8.91^a^	13.28 ± 7.16^a^	13.40 ± 6.76^a^	0.010	0.990
	6 M follow-up	10.74 ± 5.46^a^	12.12 ± 5.58^a^	13.69 ± 6.14^a^	1.762	0.176
F value		37.176	123.751	39.609		
*p *value		**<0.001**	**<0.001**	**<0.001**		
Global text						
	group (F value, *p *value)	0.574, 0.565				
	time (F value, *p *value)	286.727, **<0.001**				
	group*time (F value, *p *value)	2.544, 0.077				
AVA (cm^2^, *x̅* ± *s)*						
	Baseline	0.75 ± 0.15	0.70 ± 0.20	0.70 ± 0.25	0.468	0.627
	Discharge	1.77 ± 0.51^a^	1.63 ± 0.43^a^	1.58 ± 0.35^a^	1.326	0.269
	6 M follow-up	1.86 ± 0.33^a^	1.72 ± 0.37^ab^	1.64 ± 0.38^a^	2.301	0.105
F value		80.040	242.477	104.965		
*p *value		**<0.001**	**<0.001**	**<0.001**		
Global text						
	group (F value, *p *value)	2.085, 0.129				
	time (F value, *p *value)	434.409, **<0.001**				
	group*time (F value, *p *value)	0.838, 0.493				
AVA/BSA (cm^2^/m^2^, *x̅* ± *s*)						
	Baseline	0.41 ± 0.08	0.39 ± 0.12	0.39 ± 0.13	0.210	0.811
	Discharge	0.96 ± 0.25^a^	0.91 ± 0.21^a^	0.97 ± 0.19^a^	0.915	0.403
	6 M follow-up	1.02 ± 0.16^a^	0.89 ± 0.16^ab^	0.93 ± 0.19^a^	1.366	0.259
F value		80.849	258.811	117.097		
*p *value		**<0.001**	**<0.001**	**<0.001**		
Global text						
	group (F value, *p *value)	1.423, 0.245				
	time (F value, *p *value)	447.817, **<0.001**				
	group*time (F value, *p *value)	0.454, 0.761				
LVEF (%, *x̅* ± *s*)						
	Baseline	56.32 ± 10.64	52.93 ± 11.97	49.26 ± 13.74	2.161	0.120
	Discharge	58.68 ± 8.35	55.49 ± 9.48^a^	52.71 ± 11.58^a^	2.275	0.107
	6 M follow-up	59.63 ± 8.15	58.34 ± 7.57^ab^	57.03 ± 10.53^ab^	0.531	0.590
F value		1.251	9.890	11.040		
*p *value		0.290	**<0.001**	**<0.001**		
Global text						
	group (F value, *p *value)	1.991, 0.141				
	time (F value, *p *value)	22.245, **<0.001**				
	group*time (F value, *p *value)	1.187, 0.316				
LVEDD (mm, *x̅* ± *s*)						
	Baseline	50.00 ± 6.98	51.43 ± 8.24	52.26 ± 8.19	0.485	0.617
	Discharge	48.95 ± 5.31	49.94 ± 7.19^a^	52.26 ± 7.54	1.761	0.176
	6 M follow-up	47.32 ± 5.40	48.40 ± 6.59^ab^	49.26 ± 7.45^ab^	0.530	0.590
F value		2.120	10.564	7.964		
*p *value		0.125	**<0.001**	**<0.001**		
Global text						
	group (F value, *p *value)	0.904, 0.408				
	time (F value, *p *value)	20.213, **<0.001**				
	group*time (F value, *p *value)	0.841, 0.470				
RAD (mm, *x̅* ± *s*)						
	Baseline	29.68 ± 5.68	47.40 ± 9.18^A^	46.14 ± 8.53^A^	33.044	**<0.001**
	Discharge	27.74 ± 5.33	45.07 ± 9.10^aA^	44.80 ± 8.49^A^	33.361	**<0.001**
	6 M follow-up	28.84 ± 4.30	45.41 ± 9.14^aA^	44.29 ± 8.42^aA^	30.165	**<0.001**
F value		2.074	9.896	3.292		
*p *value		0.130	**<0.001**	**0.041**		
Global text						
	group (F value, *p *value)	34.783, **<0.001**				
	time (F value, *p *value)	11.614, **<0.001**				
	group*time (F value, *p *value)	0.787, 0.527				
RVDD (mm, *x̅* ± *s*)						
	Baseline	32.79 ± 3.51	47.43 ± 9.97^A^	50.31 ± 13.72^A^	18.390	**<0.001**
	Discharge	32.26 ± 3.56	46.43 ± 9.63^A^	48.31 ± 12.55^aA^	18.150	**<0.001**
	6 M follow-up	31.63 ± 2.19	46.60 ± 9.44^A^	47.77 ± 12.50^aA^	19.990	**<0.001**
F value		0.795	2.944	7.648		
*p *value		0.454	0.057	**<0.001**		
Global text						
	group (F value, *p *value)	19.532, **<0.001**				
	time (F value, *p *value)	9.558, **<0.001**				
	group*time (F value, *p *value)	1.624, 0.175				
AAD (mm, *x̅* ± *s*)						
	Baseline	20.95 ± 2.01	20.63 ± 2.66	20.03 ± 2.24	1.058	0.350
	Discharge	19.84 ± 2.29	20.16 ± 2.48	19.60 ± 3.27	0.517	0.598
	6 M follow-up	19.42 ± 1.50^a^	19.90 ± 2.51^a^	19.14 ± 2.35	1.276	0.283
F value		4.164	3.436	2.679		
*p *value		**0.018**	**0.035**	0.073		
Global text						
	group (F value, *p *value)	1.041, 0.356				
	time (F value, *p *value)	10.398, **<0.001**				
	group*time (F value, *p *value)	0.559, 0.692				
LVPWT (mm, *x̅* ± *s*)						
	Baseline	11.84 ± 2.46	12.34 ± 2.06	11.66 ± 2.00	1.330	0.268
	Discharge	11.68 ± 2.08	11.99 ± 1.77^a^	11.63 ± 1.66	0.540	0.584
	6 M follow-up	10.74 ± 1.24^ab^	11.49 ± 1.52^ab^	11.11 ± 1.32^b^	2.308	0.104
F value		7.276	11.826	3.704		
*p *value		**0.001**	**<0.001**	**0.028**		
Global text						
	group (F value, *p *value)	1.316, 0.272				
	time (F value, *p *value)	23.001, **<0.001**				
	group*time (F value, *p *value)	1.058, 0.376				
IVSD (mm, *x̅* ± *s*)						
	Baseline	13.63 ± 2.17	13.57 ± 2.59	12.60 ± 2.16	2.093	0.128
	Discharge	13.26 ± 2.38	13.29 ± 2.26	12.46 ± 1.88	1.815	0.167
	6 M follow-up	12.47 ± 1.84^ab^	12.60 ± 2.02^ab^	11.86 ± 1.73^ab^	1.782	0.173
F value		4.240	10.875	3.633		
*p *value		**0.017**	**<0.001**	**0.029**		
Global text						
	group (F value, *p *value)	2.195, 0.116				
	time (F value, *p *value)	22.384, **<0.001**				
	group*time (F value, *p *value)	0.285, 0.845				
LVMi (g/m^2^, *x̅* ± *s*)						
	Baseline	141.85 ± 40.99	153.79 ± 41.20	146.38 ± 41.55	0.792	0.455
	Discharge	132.46 ± 34.19	141.85 ± 33.82^a^	142.98 ± 33.58	0.684	0.507
	6 M follow-up	113.56 ± 28.79^ab^	126.23 ± 30.02^ab^	122.05 ± 29.24^ab^	1.386	0.254
F value		9.971	32.375	17.992		
*p *value		**<0.001**	**<0.001**	**<0.001**		
Global text						
	group (F value, *p *value)	0.905, 0.407				
	time (F value, *p *value)	67.949, **<0.001**				
	group*time (F value, *p *value)	0.862, 0.465				
RWT (*x̅* ± *s*)						
	Baseline	0.52 ± 0.13	0.52 ± 0.13	0.48 ± 0.12	1.226	0.297
	Discharge	0.52 ± 0.12	0.52 ± 0.12	0.47 ± 0.11	1.992	0.141
	6 M follow-up	0.49 ± 0.06	0.51 ± 0.10	0.48 ± 0.10	1.118	0.330
F value		1.001	0.659	0.386		
*p *value		0.371	0.519	0.680		
Global text						
	group (F value, *p *value)	1.609, 0.204				
	time (F value, *p *value)	1.577, 0.213				
	group*time (F value, *p *value)	0.571, 0.647				
mPAD (mm, *x̅* ± *s*)						
	Baseline	24.00 ± 2.85	26.66 ± 4.16^A^	26.00 ± 3.89	3.447	**0.035**
	Discharge	23.84 ± 3.18	25.51 ± 3.70	25.51 ± 3.98	1.630	0.200
	6 M follow-up	23.68 ± 2.31	25.29 ± 3.79	25.71 ± 4.46	1.827	0.165
F value		0.046	4.728	0.385		
*p *value		0.955	0.074	0.681		
Global text						
	group (F value, *p *value)	3.371, **0.038**				
	time (F value, *p *value)	1.514, 0.224				
	group*time (F value, *p *value)	0.594, 0.650				
mPAD/BSA (mm, *x̅* ± *s*)						
	Baseline	13.12 ± 1.47	15.00 ± 2.56^A^	14.86 ± 3.04	4.076	**0.019**
	Discharge	13.06 ± 1.85	14.38 ± 2.48	14.51 ± 2.69	2.528	0.084
	6 M follow-up	12.98 ± 1.53	14.22 ± 2.30	14.59 ± 2.82	2.929	0.057
F value		0.024	4.250	0.588		
*p *value		0.976	0.067	0.557		
Global text						
	group (F value, *p *value)	4.334, **0.015**				
	time (F value, *p *value)	1.596, 0.207				
	group*time (F value, *p *value)	0.492, 0.723				

PASP, pulmonary artery systolic pressure; AR, aortic regurgitation; MR, mitral regurgitation; TR, tricuspid regurgitation; MPG, mean aortic gradient; AVA, aortic valve area; LVEF, left ventricular ejection fraction; LVEDD, left ventricular end-diastolic dimension; RAD, right atrial diameter; RVD, right ventricular diameter; RVDD, right ventricular diastolic diameter; AAD, aortic valve annulus diameter; LVPWT, left ventricular posterior wall thickness; IVSD, interventricular septum in diastole; LVMi, left ventricular mass index; RWT, relative wall thickness; mPAD, main pulmonary artery diameter; BSA, body surface area; Middle: Compared with the same group at baseline, ^a^*p* < 0.05; Compared with the same group at discharge, ^b^*p* < 0.05, *p* values in bold are statistically significant; Middle: Compared with the No PH group at the same follow-up time, ^A^*p* < 0.05, Compared with the Mild-to-moderate PH group at the same follow-up time, ^B^*p* < 0.05, *p* values in bold are statistically significant; Down (*p* value): Compared with the same group in total (at baseline, discharge or 6 M follow-up), *p* values in bold are statistically significant in the same group; Down (Global text: group: *p* value): Compared with the No PH, Mild-to-moderate PH and Severe PH groups in total (at baseline, discharge or 6 M follow-up), *p* < 0.05; Down (Global text: time: *p* value): Compared with each follow-up time, *p* < 0.05; Down (Global text: group*time: *p* value): Compared with each follow-up time, *p* > 0.05; not significant in the interaction between groups and time; If the sphericity assumption was violated, the conservative Greenhouse–Geisser correction was applied; adjustments for multiple pairwise comparisons were applied using the Bonferroni correction.

For right ventricular hemodynamic parameters in three groups, at baseline, PASP increased progressively across groups (No PH, mild-to-moderate PH, and severe PH; all *p* < 0.05). Specifically, compared with the No PH group, PASP was significantly higher in both the mild-to-moderate PH and severe PH groups (^A^*p* < 0.05), and was further elevated in the severe PH group compared with the mild-to-moderate PH group (^B^*p* < 0.05). Similar between-group differences were observed at discharge and at 6-month follow-up (all *p* < 0.05).

For right ventricular hemodynamic parameters at different follow-up times, PASP remained stable over time in the No PH group (*p *= 0.844) following TAVI. In contrast, significant temporal changes were observed in both the mild-to-moderate and severe PH groups (both *p* < 0.001). Post hoc comparisons demonstrated that PASP was significantly reduced at discharge compared with baseline in the PH groups (^a^*p *< 0.05), with additional changes observed between discharge and 6-month follow-up (^b^*p* < 0.05).

#### 3.3.2 Left Ventricular Hemodynamic Parameters

In contrast, left ventricular hemodynamic parameters, including MPG, AVA, and AVA/BSA, showed significant improvement over time in all groups (all *p* < 0.001), without significant differences between groups or group*time interaction, suggesting a uniform hemodynamic benefit of TAVI on valvular obstruction, as shown in Table 3.

#### 3.3.3 Cardiac Structural Parameters

Structural cardiac parameters, including left ventricular dimensions and mass indices, demonstrated gradual improvement over time (all *p* < 0.001 for time effect), whereas right heart dimensions (right atrial diameter [RAD] and right ventricular diastolic diameter [RVDD]) remained significantly larger in PH groups throughout follow-up (all *p* < 0.001 for group effect), with limited reverse remodeling (Table 3).

#### 3.3.4 Pulmonary Artery Structural Parameters

Pulmonary artery diameter (mPAD and mPAD/BSA) was significantly higher in PH groups at baseline (*p* < 0.05), but did not show significant changes over time or interaction effects, indicating relative stability of vascular structural changes after TAVI (Table 3).

**Fig. 2.**
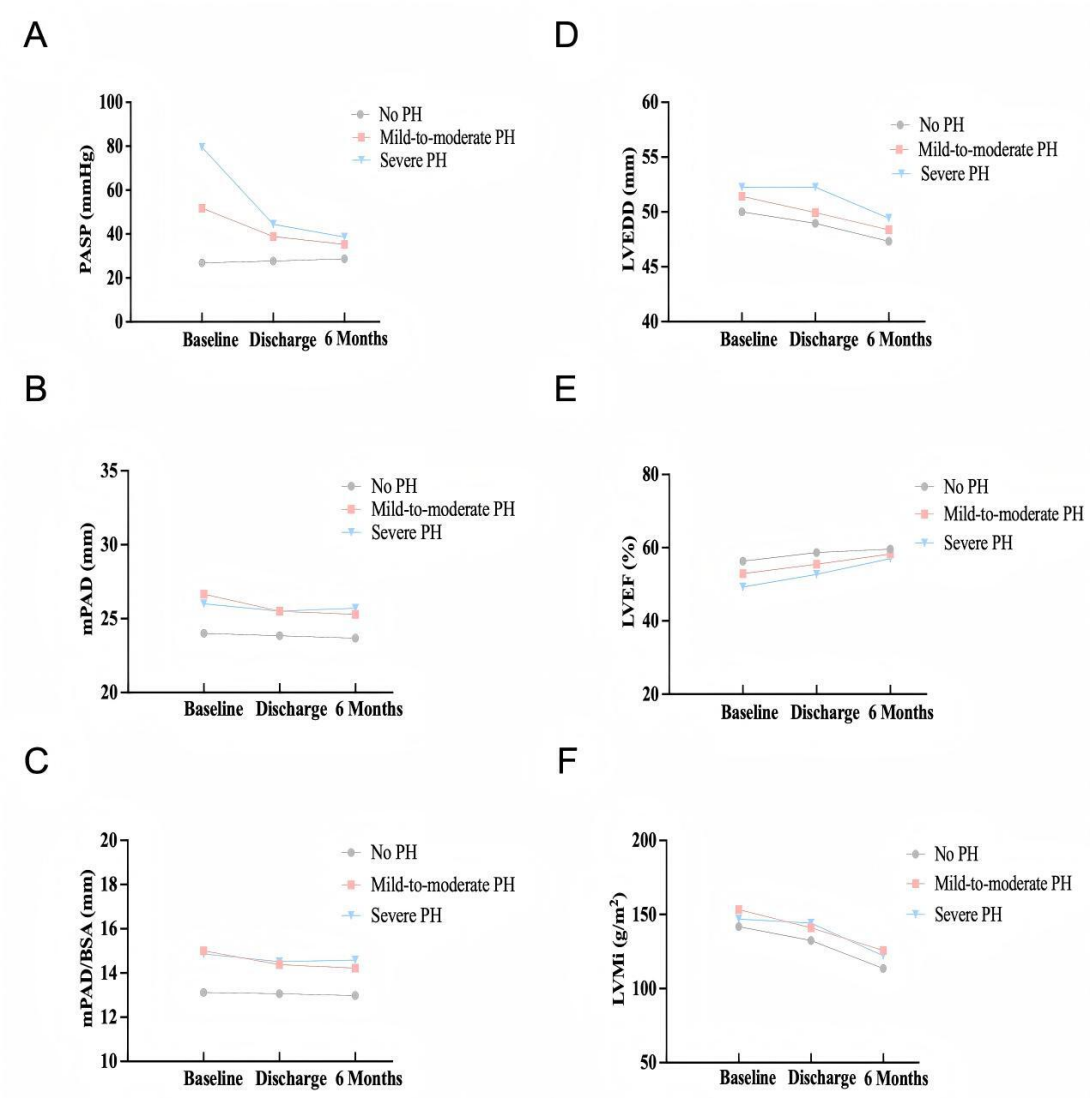
**Comparison of pre- and post-TAVI echocardiography and MDCT parameters in patients with or without PH**. Changes of (A) pulmonary artery systolic pressure, (B) main pulmonary artery diameter; (C) pulmonary artery diameter index to body surface area; (D) left ventricular end-diastolic dimension; (E) left ventricular ejection fraction; (F) left ventricular mass index in patients with no PH, mild-to-moderate PH and severe PH patients. PASP, pulmonary artery systolic pressure; mPAD, main pulmonary artery diameter; mPAD/BSA, main pulmonary artery diameter index to body surface area; LVEDD, left ventricular end-diastolic dimension; LVEF, left ventricular ejection fraction; LVMi, left ventricular mass index; TAVI, transcatheter aortic valve implantation; PH, pulmonary hypertension; MDCT, multidetector computed tomography.

In contrast, mPAD assessed by MDCT did not show significant changes over time, suggesting that vascular remodeling may lag behind hemodynamic improvement in patients with severe PH. Between-group comparisons revealed greater PASP reduction in severe PH compared with mild-to-moderate PH and no-PH groups (*p* < 0.05), whereas reductions in mPAD and mPAD/BSA were more pronounced in mild-to-moderate PH (*p* < 0.05), as shown in Fig. 2.

#### 3.3.5 Comparison of Echocardiographic and MDCT Parameters Between PASP Improvement and Non-Improvement Groups

**Fig. 3.**
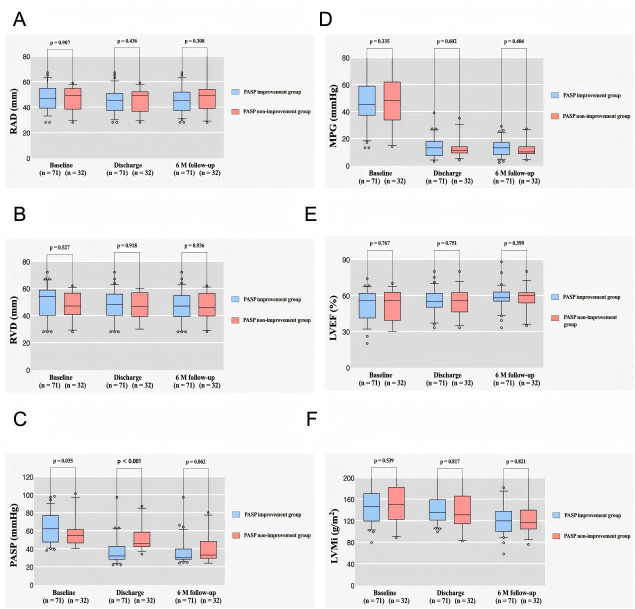
**Comparison of echocardiography and MDCT parameters in patients with or without PASP improvement**. Changes of (A) right atrial diameter, (B) right ventricular diameter; (C) pulmonary artery systolic pressure; (D) mean transvalvular pressure gradient; (E) left ventricular ejection fraction; (F) left ventricular mass index in patients of PASP improvement and non-improvement group. RAD, right atrial diameter; RVD, right ventricular diameter; PASP, pulmonary artery systolic pressure; MPG, mean transvalvular pressure gradient; LVEF, left ventricular ejection fraction; LVMi, left ventricular mass index; PH, pulmonary hypertension; MDCT, multidetector computed tomography.

As shown in Fig. 3, patients were stratified according to postoperative PASP reduction (Yes, n = 71; No, n = 32). At baseline, PASP was significantly higher in the PASP improvement group compared with the non-improvement group (63.31 ± 16.44 vs. 56.50 ± 14.08 mmHg, *p *= 0.035). Following the procedure, PASP decreased markedly in the improvement group at discharge and remained lower at 6-month follow-up, whereas PASP remained persistently elevated in the non-improvement group (both *p* < 0.001 at discharge; *p* = 0.062 at 6 months).

In contrast, no significant differences were observed between the two groups in RAD, RVD, MPG, LVEF, or LVMi at baseline, discharge, or 6-month follow-up (all *p* > 0.05). Both groups demonstrated comparable improvements in transvalvular hemodynamics and left ventricular systolic function after TAVI, accompanied by progressive reductions in LVMi over time.

Collectively, these findings indicate that patients with postoperative PASP improvement had higher baseline pulmonary pressures and exhibited greater PASP reduction after TAVI. Notably, however, structural right heart dimensions and left ventricular remodeling parameters evolved similarly in both the PASP improvement and no improvement groups. This dissociation suggests that pulmonary pressure reversibility may occur independently of short-term changes in global cardiac structure, supporting the concept that persistent pulmonary vascular remodeling contributes to PASP non-improvement in a subset of patients.

### 3.4 Factors Associated With Postoperative PASP Non-Improvement

**Fig. 4.**
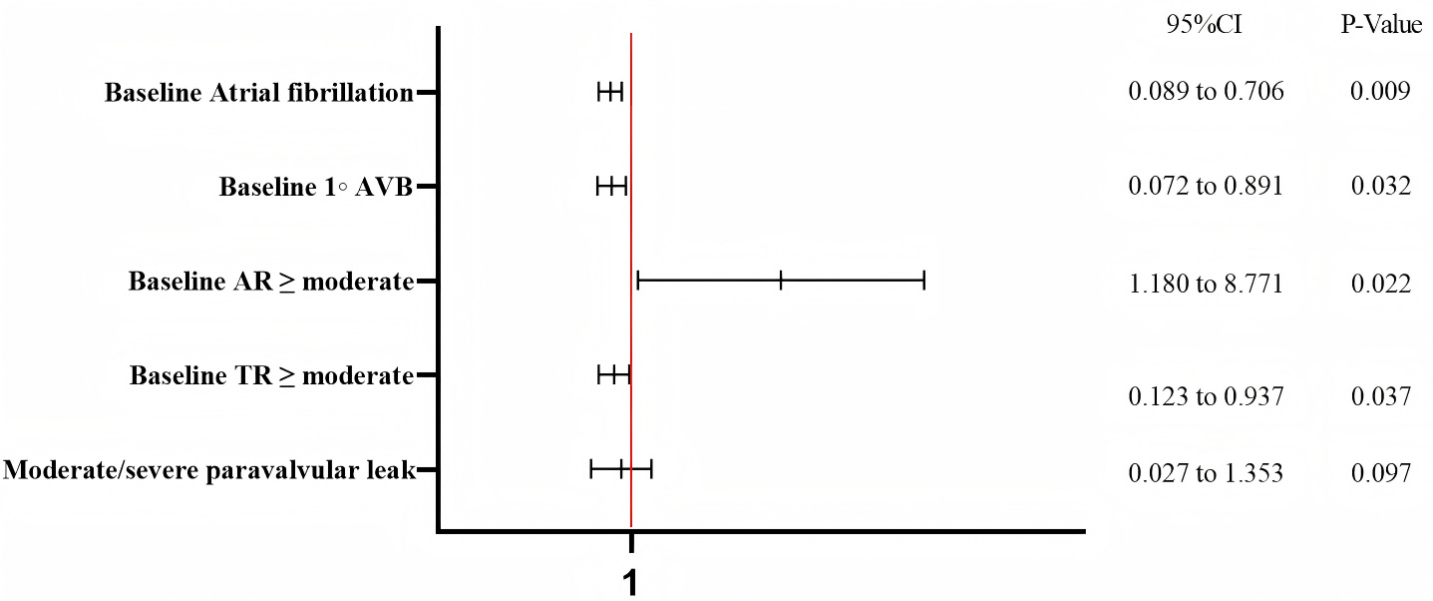
**Independent predictors of PASP non-improvement post-TAVI**. Symbols represent point estimates (odds ratios [ORs]), and horizontal lines denote 95% confidence intervals derived from multivariable logistic regression. 1° AVB, first-degree atrioventricular block; AR, aortic regurgitation; TR, tricuspid regurgitation.

In multivariable logistic regression analysis (Table 4, Fig. 4), baseline atrial fibrillation (OR 0.251, 95% CI 0.089–0.706, *p* = 0.009), preoperative first-degree atrioventricular block (OR 0.253, 95% CI 0.072–0.891, *p* = 0.032), and preoperative ≥ moderate tricuspid regurgitation (OR 0.339, 95% CI 0.123–0.937, *p* = 0.037) were independently associated with a lower risk of postoperative PASP non-improvement, whereas preoperative ≥ moderate aortic regurgitation was associated with a higher risk of PASP non-improvement (OR 3.217, 95% CI 1.180–8.771, *p* = 0.022).

**Table 4.** **Independent predictors of PASP non-improvement post-TAVI**.

	Beta	OR	95% CI	*p-*value
Baseline Atrial fibrillation	–1.381	0.251	0.089 to 0.706	**0.009**
Baseline 1° AVB	–1.374	0.253	0.072 to 0.891	**0.032**
Baseline AR ≥ moderate	1.169	3.217	1.180 to 8.771	**0.022**
Baseline TR ≥ moderate	–1.081	0.339	0.123 to 0.937	**0.037**
Moderate/severe paravalvular leak	–1.656	0.191	0.027 to 1.353	0.097

Adjusted for BAV, Hypertension, Dyslipidemia, RBBB, AAD, 1° AVB (first-degree atrioventricular block), AR (aortic regurgitation), and TR (tricuspid regurgitation). *p* values in bold are statistically significant.

In sensitivity analyses using alternative definitions of PASP change, the results were not entirely consistent with those of the primary categorical endpoint. Baseline ≥ moderate aortic regurgitation showed a directionally consistent association across models, being associated with a higher risk of PASP non-improvement in the logistic regression analysis and with a smaller reduction (or greater increase) in PASP in the linear regression analysis. However, other variables did not demonstrate consistent associations across different analytical approaches, as shown in **Supplementary Tables 1,2**.

### 3.5 One-Year All-Cause and Cardiac Mortality: Kaplan–Meier and Cox Regression Analyses

**Fig. 5.**
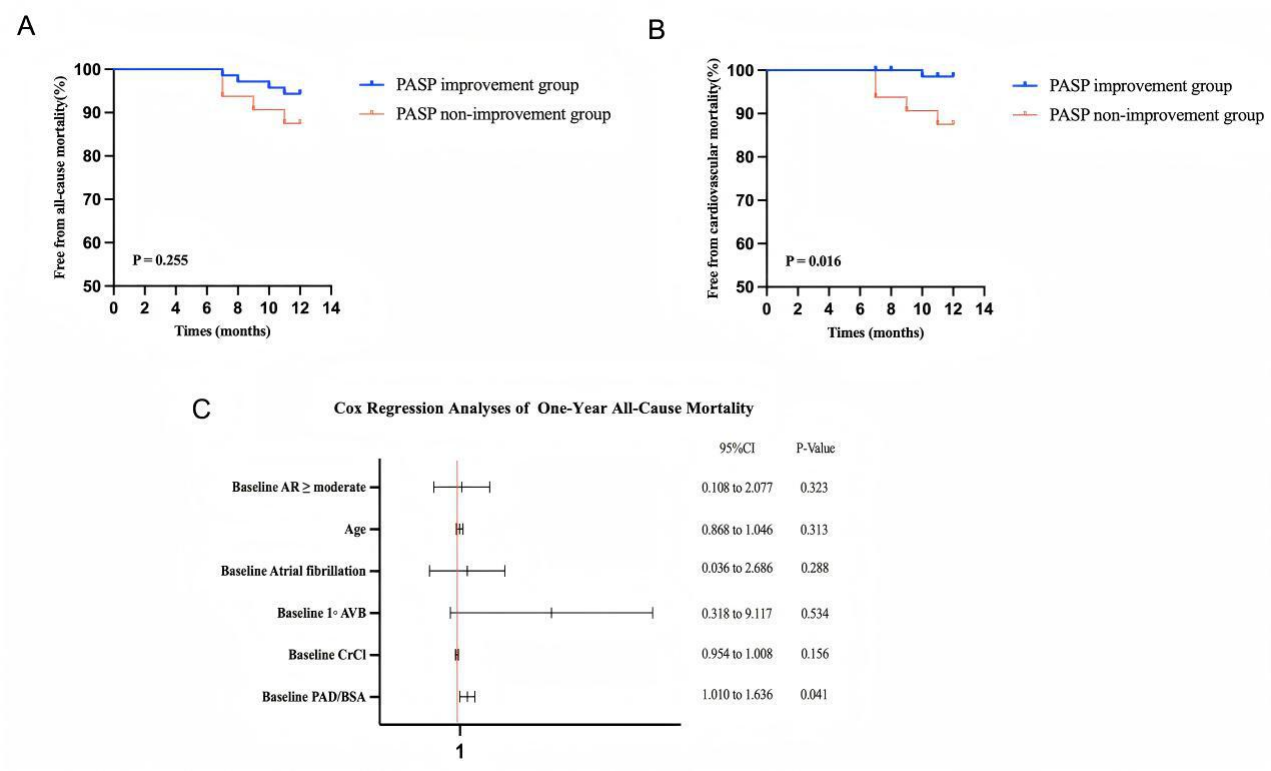
**One-Year All-Cause and Cardiac Mortality: Kaplan–Meier and Cox Regression Analyses**. (A) Free from all-cause mortality; (B) Free from cardiac mortality; (C) Cox regression analyses of one-year mortality. AR, aortic regurgitation; TR, tricuspid regurgitation; 1° AVB, first-degree atrioventricular block; CrCl, creatinine clearance; mPAD/BSA, particularly main pulmonary artery diameter index to body surface area.

During the 1-year follow-up, a total of 8 all-cause deaths (7.7%), including 5 cardiovascular deaths (4.9%), occurred, and these events were included in the Cox regression analysis. As shown in Fig. 5, Kaplan–Meier survival analysis demonstrated no significant difference in 1-year all-cause mortality between patients with and without postoperative PASP improvement (log-rank *p* = 0.255). In multivariable Cox proportional hazards analysis (Table 5), preoperative mPAD/BSA was independently associated with 1-year all-cause mortality (HR 1.29, 95% CI 1.01–1.64, *p* = 0.041).

**Table 5.** **Cox Regression Analyses of one-year all-cause mortality**.

	Model 1			Model 2		
	Beta	95% CI	*p* value	Beta	95% CI	*p* value
Baseline AR ≥ moderate	–0.370	0.173 to 2.763	0.601	–0.745	0.108 to 2.077	0.323
Age	–0.023	0.891 to 1.070	0.615	–0.048	0.868 to 1.046	0.313
Baseline Atrial fibrillation	–1.135	0.040 to 2.613	0.288	–1.172	0.036 to 2.686	0.288
Baseline 1° AVB	0.561	0.353 to 8.680	0.492	0.532	0.318 to 9.117	0.534
Baseline CrCl	–0.020	0.956 to 1.005	0.110	–0.020	0.954 to 1.008	0.156
Baseline mPAD/BSA	0.285	1.053 to 1.679	**0.017**	0.251	1.010 to 1.636	**0.041**

Model 1: Univariable Cox regression analysis. Model 2: Multivariable Cox regression analysis adjusted for bicuspid aortic valve (BAV), hypertension, dyslipidemia, right bundle branch block (RBBB), and aortic aneurysm/dissection (AAD). AR, aortic regurgitation; 1° AVB, first-degree atrioventricular block; CrCl, creatinine clearance; mPAD/BSA, main pulmonary artery diameter index to body surface area. The C-statistic for all-cause mortality was 0.828. *p*-values in bold indicate statistical significance.

As shown in Fig. 5, Kaplan–Meier analysis demonstrated a significantly lower 1-year cardiac mortality in patients with postoperative PASP improvement compared with those without improvement (log-rank *p* = 0.016). In multivariable Cox regression analysis for 1-year cardiac mortality (Table 6), none of the included variables, including preoperative main pulmonary artery diameter, ≥ moderate aortic regurgitation, atrial fibrillation, creatinine clearance, preoperative conduction disturbances, or age, showed an independent association with cardiac mortality.

**Table 6.** **Cox Regression Analyses of one-year cardiac mortality**.

	Model 1			Model 2		
	Beta	95% CI	*p*-value	Beta	95% CI	*p*-value
Baseline AR ≥ moderate	–0.770	0.077 to 2.771	0.399	–1.104	0.046 to 2.405	0.275
Age	0.007	0.893 to 1.131	0.932	–0.029	0.863 to 1.093	0.627
Baseline Atrial fibrillation	–0.578	0.063 to 5.022	0.605	–0.940	0.034 to 4.455	0.449
Baseline 1° AVB	1.254	0.585 to 20.978	0.170	1.396	0.551 to 29.635	0.170
Baseline CrCl	–0.027	0.942 to 1.006	0.106	–0.029	0.934 to 1.010	0.141
Baseline mPAD/BSA	0.319	1.021 to 1.853	**0.036**	0.270	0.969 to 1.771	0.079

Model 1: Univariable Cox regression analysis. Model 2: Multivariable Cox regression analysis adjusted for bicuspid aortic valve (BAV), hypertension, dyslipidemia, right bundle branch block (RBBB), and aortic aneurysm/dissection (AAD). AR, aortic regurgitation; 1° AVB, first-degree atrioventricular block; CrCl, creatinine clearance; mPAD/BSA, particularly main pulmonary artery diameter index to body surface area. The C-statistic for cardiovascular mortality was 0.814. *p* values in bold indicate statistical significance.

No significant violations of the proportional hazards assumption were observed for any covariates (all *p* > 0.05), as shown in **Supplementary Tables 3,4**. The C-statistic for the prediction of 1-year all-cause mortality was 0.828, indicating good model discrimination. Similarly, the C-statistic for cardiovascular mortality was 0.814. The inclusion of mPAD/BSA improved model discrimination beyond PASP alone.

## 4. Discussion

In this study, we investigated pulmonary hemodynamic changes and clinical outcomes after TAVI across different severities of PH. Several important findings emerged (1) patients in the PH improvement group had significantly smaller mPAD and mPAD/BSA compared with those in the non-improvement group before TAVI procedures; (2) moderate or severe paravalvular leak occurred less frequently in the PH improvement group than in the non-improvement group; (3) significant improvements in PASP were observed in patients with pre-existing PH, the magnitude and trajectory of recovery differed according to baseline severity, with persistently elevated pressures in more advanced cases; (4) left ventricular hemodynamics improved uniformly across all groups, whereas right heart remodeling and pulmonary artery structural changes remained limited; (5) multivariable logistic regression analysis demonstrated that preoperative ≥ moderate aortic regurgitation was independently associated with postoperative PASP non-improvement; (6) cox analysis revealed that preoperative mPAD/BSA emerged as an independent predictor of 1-year all-cause mortality, highlighting the prognostic relevance of pulmonary vascular remodeling beyond PASP alone; (7) Kaplan–Meier analysis demonstrated a significantly lower 1-year cardiac mortality in patients with postoperative PASP improvement compared with those without improvement. Collectively, these findings suggest that PH secondary to AS is partially reversible after TAVI, particularly at earlier stages. Moreover, they highlight the importance of comprehensive pulmonary vascular and right heart assessment for risk stratification and timing of intervention.

### 4.1 Pulmonary Hypertension Assessment in the TAVI Population: Structural and Hemodynamic Perspectives

Previous studies have shown that PH serves as a strong prognostic indicator in patients undergoing TAVI, particularly when evaluated using invasive hemodynamic assessment [24]. In contrast to prior studies that primarily rely on invasive hemodynamic data, our study focused on structural parameters of the pulmonary artery derived from MDCT. These approaches may offer complementary insights, as structural remodeling reflects chronic adaptation within the pulmonary circulation, whereas hemodynamic measurements reflect dynamic pressure loading conditions.

Taken together, these findings suggest that integrating structural imaging markers with hemodynamic assessment may improve risk stratification in patients undergoing TAVI. However, as our study relied on echocardiography-derived PASP rather than invasive hemodynamic measurements, these findings should be interpreted with caution and warrant further validation.

The present study demonstrates that TAVI leads to significant improvements in left ventricular hemodynamics, as reflected by reductions in transvalvular gradients and increases in aortic valve area, regardless of baseline pulmonary hypertension status. However, the response of PASP was heterogeneous across groups, with significant reductions observed only in patients with pre-existing PH. Notably, the significant group*time interaction for PASP suggests that the magnitude and trajectory of pulmonary pressure improvement depend on baseline PH severity. This finding supports the concept that elevated PASP in aortic stenosis is, at least partially, driven by reversible post-capillary mechanisms related to left-sided pressure overload, which can be alleviated after TAVI.

Despite these improvements, PASP remained persistently elevated in PH groups, and right heart structural parameters showed limited reverse remodeling. This may reflect chronic pulmonary vascular remodeling and increased pulmonary vascular resistance, which are less responsive to relief of valvular obstruction alone. In contrast, mPAD did not significantly change over time, further suggesting that vascular structural alterations may represent more advanced and less reversible disease.

Together, these findings indicate that while TAVI effectively improves valvular hemodynamics, pulmonary vascular and right heart remodeling may lag behind, particularly in patients with established PH.

### 4.2 Clinical Relevance of mPAD Indexed to Body Surface Area

In the present study, we used mPAD/BSA instead of alternative metrics, including the pulmonary artery-to-aorta (PA/Ao) ratio [25]. Although widely used, the PA/Ao ratio is a relative parameter and may be influenced by variations in aortic size, which are associated with factors such as age, hypertension, and degenerative changes commonly observed in patients with aortic stenosis [26,27]. Therefore, it may not reliably reflect pulmonary arterial remodeling in this population.

In contrast, indexing mPAD to BSA yields a body size–adjusted metric that accounts for interindividual variability in body habitus, thereby enhancing comparability across patients. This approach enables a more direct evaluation of pulmonary arterial enlargement independent of aortic size. Given that patients undergoing TAVI are typically elderly and demonstrate considerable variability in body size and vascular characteristics, mPAD/BSA may serve as a more physiologically relevant structural marker.

Importantly, our findings indicate that preoperative mPAD/BSA is an independent predictor of 1-year all-cause mortality beyond PASP, supporting its potential as a novel imaging biomarker. The integration of MDCT-derived structural parameters with echocardiographic evaluation may further improve risk stratification and facilitate clinical decision-making in patients undergoing TAVI.

In our study, pulmonary artery systolic pressure was estimated using echocardiography rather than invasive hemodynamic measurements. Although widely used in clinical practice, PASP derived from tricuspid regurgitation velocity represents a non-invasive surrogate and cannot distinguish between isolated post-capillary pulmonary hypertension and combined post- and pre-capillary disease. As a result, the mechanistic interpretation of pulmonary vascular remodeling and its reversibility after TAVI should be made with caution. In the present study, mPAD/BSA was evaluated in relation to echocardiography-derived PASP rather than direct measurements of pulmonary vascular physiology. Therefore, our findings primarily reflect associations with non-invasive hemodynamic estimates rather than definitive characterization of pulmonary vascular disease.

### 4.3 Pulmonary Vascular Remodeling May Lag Behind Hemodynamic Improvement in Advanced Disease

Our study demonstrated that structural mPAD does not necessarily parallel hemodynamic improvement, particularly in advanced or long-standing PH disease. Our findings further underscore a dissociation between hemodynamic improvement and structural pulmonary arterial remodeling. We selected discharge PASP as the primary time point because it reflects the early hemodynamic response to TAVI. Relief of left ventricular outflow obstruction following valve implantation leads to an immediate reduction in left ventricular filling pressure and pulmonary venous congestion, which in turn results in an early decrease in pulmonary artery pressure. Therefore, discharge PASP provides a direct assessment of the acute hemodynamic effect of the procedure. In contrast, longer-term PASP measurements may be influenced by a variety of postprocedural factors, including changes in medical therapy, volume status, right ventricular remodeling, and comorbid conditions. As such, discharge PASP is less confounded by these factors and may better reflect the intrinsic response of the pulmonary circulation to TAVI. In our cohort, the extent and profile of pulmonary and cardiac responses varied with baseline PH severity. MDCT investigations have shown that longitudinal alterations in main pulmonary artery caliber progress slowly and can trail clinical or hemodynamic improvement, indicating that pulmonary arterial size serves as a comparatively delayed structural indicator of disease activity.

Tonelli et al. [28] observed that longitudinal variation in main pulmonary artery caliber correlated with disease trajectory, RV performance, and clinical outcomes, suggesting that mPAD functions as a delayed structural indicator that does not necessarily mirror short-term pressure changes. Similarly, Boerrigter et al. [29] demonstrated progressive pulmonary artery enlargement over follow-up and its relationship with post-treatment hemodynamics, further indicating that vascular remodeling can trail reductions in pulmonary arterial pressure. In parallel, prior CT-based correlation studies have reported only modest and variable associations between pulmonary artery size and pulmonary arterial pressures, highlighting the nonlinear relationship and substantial interindividual heterogeneity between vascular morphology and hemodynamics [30]. Furthermore, contemporary imaging reviews highlight that pulmonary artery enlargement represents cumulative vascular remodeling rather than acute pressure burden, thereby offering a pathophysiologic basis for understanding discrepant changes between PASP and pulmonary artery size [31].

Consistent with prior reports describing a dissociation between hemodynamic improvement and structural pulmonary vascular changes, our findings further highlight this phenomenon in advanced disease. This dissociation suggests that pulmonary vascular remodeling may trail hemodynamic improvement in advanced disease, possibly reflecting fixed pulmonary vascular alterations. These findings lend support to the premise that earlier intervention may provide greater potential for pulmonary vascular reversibility.

### 4.4 Influence of Right-Sided Cardiac Dysfunction and Electrical Conduction Abnormalities on Pulmonary Pressure Reversibility

Our study demonstrated that AF, preoperative first-degree AVB, and preoperative ≥ moderate TR were independently linked to greater PASP improvement after the procedure. AF has been associated with chronic right atrial pressure overload and structural remodeling, and in the setting of pulmonary hypertension, it is often linked to more advanced disease and adverse outcomes. Moreover, AF and flutter have been strongly associated with right atrial enlargement and RV dysfunction, suggesting a persistent hemodynamic load on the right heart [32].

Within the TAVI population, Barbanti et al. [33] reported that preoperative moderate or greater TR was associated with persistent pulmonary hypertension and worse clinical outcomes. The discrepancy between their findings and ours may be related to differences in patient characteristics, study design, and definitions of pulmonary pressure response.

Such chronic structural and functional alterations may be associated with differences in pulmonary pressure response following relief of aortic stenosis. These findings highlight the potential role of right-sided cardiac dysfunction and conduction disturbances in pulmonary pressure response. In the present study, these factors were associated with greater PASP improvement, suggesting that baseline right-sided cardiac and conduction abnormalities may be related to differences in pulmonary pressure response following TAVI. However, given that PASP was assessed using echocardiography rather than invasive hemodynamic measurements, these findings should be interpreted as associations rather than direct evidence of pulmonary vascular remodeling.

### 4.5 Preoperative mPAD/BSA Emerged as an Independent Predictor of 1-Year All-Cause Mortality

Survival analyses demonstrated divergent trajectories for all-cause versus cardiac mortality. While postoperative improvement of PASP correlated with reduced one-year cardiac mortality in Kaplan–Meier analysis, no independent determinants of cardiac death emerged in multivariable Cox regression, likely due to the small number of cardiac events. In contrast, baseline main pulmonary artery diameter indexed to body surface area independently predicted one-year all-cause mortality.

In keeping with this observation, prior studies have shown that pulmonary artery enlargement correlates with PH among patients treated with TAVI [6,25,34,35]. Mitsumasa S et al. [27] examined CT-derived PAD and related indices, together with their clinical relevance, in patients treated with TAVI. This result accords with prior work demonstrating a stronger correlation between PA/BSA and pulmonary artery pressure than with mPAD alone [36]. Notably, Mitsumasa S et al. [27] reported that PA/BSA outperformed the mPAD/AOD ratio for predicting PH, although mPAD/AOD has previously been evaluated in TAVI cohorts. It is conceivable that mPAD/BSA is a more precise marker for the prediction/identification of PH.

Our observation indicates that pulmonary vascular structural remodeling may confer greater prognostic value than short-term PASP variation alone, highlighting pulmonary artery enlargement as an integrative indicator of chronic pulmonary vascular load.

### 4.6 Limitations

Several limitations should be acknowledged in our study on the outcomes of TAVI procedures in PH patients. First, this was a single-center, retrospective study with a relatively modest sample size, which may limit generalizability and statistical power, particularly for cardiac mortality analyses. However, given the limited number of events, Firth’s penalized likelihood correction was applied to reduce potential bias in the Cox regression analysis, and the number of covariates included in the multivariable model was restricted to minimize the risk of overfitting. Although the proportional hazards assumption was not violated and the model demonstrated good discrimination as reflected by the C-statistic, the results should still be interpreted with caution. The association between mPAD/BSA and mortality should be interpreted with caution given the modest effect size (HR 1.29, 95% CI 1.01–1.64) and relatively wide confidence intervals. All the data were analyzed using SPSS Version 25.0 (IBM Corp., Armonk, NY, USA). The statistical analysis above may remedy such a deficiency to a large extent. We strongly believe the findings of our study are reliable. Second, PASP was estimated by TTE rather than right heart catheterization, which may introduce measurement variability despite guideline-based assessment and limit differentiation between isolated post-capillary and combined pulmonary hypertension. Accordingly, mPAD/BSA was evaluated against a non-invasive surrogate, and mechanistic interpretations regarding pulmonary vascular remodeling and reversibility should be made with caution. Future studies incorporating invasive hemodynamic assessment may help to further elucidate the relationship between pulmonary artery structure and pulmonary vascular physiology in this population. Third, although MDCT enabled detailed evaluation of pulmonary artery dimensions, structural vascular indices represent relatively slow markers of disease and may not fully capture short-term hemodynamic changes. Fourth, the follow-up duration was limited to one year, precluding assessment of longer-term pulmonary vascular remodeling and clinical outcomes. We would like to enlarge our cohort and prolong the follow-up in future studies. In addition, comprehensive right ventricular functional parameters and serial invasive hemodynamics were not systematically available. Finally, residual confounding inherent to observational analyses cannot be excluded. Larger prospective multicenter studies with longer follow-up and integrated invasive and imaging assessments are warranted to validate our findings.

## 5. Conclusion

In this consecutive cohort, we investigated changes in hemodynamic parameters and pulmonary vascular structure among Chinese patients with severe aortic stenosis undergoing isolated TAVI. The following major findings were identified: (1) In patients with AS treated with TAVI, PH shows partial reversibility, with the extent of hemodynamic and structural recovery varying by baseline PH severity. (2) While left ventricular hemodynamics improved uniformly across all groups, right heart remodeling, as well as pulmonary artery structural changes, remained limited. (3) Preoperative atrial fibrillation, first-degree AVB, and moderate or greater TR were independently associated with a lower risk of PASP non-improvement after the procedure, suggesting a greater likelihood of PASP improvement in these patients. These findings suggest that baseline right-sided cardiac and conduction characteristics may be associated with the reversibility of postoperative pulmonary pressure elevation. (4) In addition, baseline mPAD/BSA independently predicts 1-year all-cause mortality, reinforcing the prognostic value of pulmonary vascular structural remodeling beyond short-term PASP variation. Taken together, these findings suggest that earlier intervention may enhance pulmonary vascular reversibility and highlight the importance of comprehensive pulmonary vascular and right heart evaluation for risk stratification and procedural timing optimization in patients treated with TAVI.

PH secondary to severe AS shows partial reversibility following TAVI, with more pronounced hemodynamic and structural recovery in patients with mild-to-moderate disease. In contrast, pulmonary vascular remodeling seems blunted in advanced PH, suggesting a dissociation between pressure decline and structural recovery. Preoperative atrial fibrillation, first-degree AVB, and ≥ moderate TR were independently associated with greater PASP improvement after TAVI, suggesting that baseline right-sided cardiac and conduction characteristics may be related to the postoperative pulmonary pressure response. Importantly, MDCT-derived mPAD/BSA confers incremental prognostic information beyond echocardiographic PASP and independently predicts 1-year all-cause mortality, identifying pulmonary artery enlargement as a clinically meaningful marker of chronic pulmonary vascular burden. These findings support integrating pulmonary vascular imaging with echocardiography to improve risk stratification and help guide procedural timing in patients treated with TAVI.

## Data Availability

The datasets analyzed in this study are not publicly accessible because of data-sharing restrictions, but may be obtained from the corresponding authors upon reasonable request.
