# Supplementary material for: Preoperative Main Pulmonary Artery Diameter Indexed to Body Surface Area Independently Predicts Mortality After Transcatheter Aortic Valve Implantation in a Chinese Population
Source: Rev Cardiovasc Med. 2026 Jun 25;27(6):50699. doi: 10.31083/RCM50699 (PMC13339175; doi:10.31083/RCM50699)

**Supplementary Material**

**Preoperative Main Pulmonary Artery Diameter Indexed to Body Surface Area Independently Predicts Mortality After Transcatheter Aortic Valve Implantation in a Chinese Population**

**Correspondence to:**

Prof. Enze Jin, Department of Cardiology, The Fourth Affiliated Hospital of Harbin Medical University, Harbin, Heilongjiang P.R. 150000, China. Electronic address: [enzejin@163.com](mailto:enzejin@163.com)

**Supplementary Tables S1-4**

**Supplementary Figure S1**

**Supplementary Table**

**Supplementary Table 1. Independent predictors of ΔPASP (pre- to post-TAVI).**

|  | Model 1 | | |  | Model 2 | | |
| --- | --- | --- | --- | --- | --- | --- | --- |
|  | Beta | 95% CI | *p* value |  | Beta | 95% CI | *p* value |
| Baseline Atrial fibrillation | -6.232 | -13.494 to 1.031 | 0.092 |  | -6.215 | -13.389 to 0.959 | 0.089 |
| Baseline 1◦ AVB | -2.297 | -11.387 to 6.793 | 0.617 |  | -1.699 | -10.569 to 7.172 | 0.705 |
| Baseline AR ≥ moderate | 7.014 | 0.305 to 13.724 | **0.041** |  | 7.077 | 0.388 to 13.765 | **0.038** |
| Baseline TR ≥ moderate | -2.463 | -9.297 to 4.370 | 0.476 |  | -1.457 | -8.372 to 5.457 | 0.677 |
| Moderate/severe paravalvular leak | -11.759 | -24.981 to 1.463 | 0.081 |  | -10.869 | -24.321 to 2.583 | 0.112 |

Model 1. Crude analysis; Model 2. Adjusted for BAV, Hypertension, Dyslipidemia, RBBB, AAD; 1◦ AVB, first-degree atrioventricular block; AR, aortic regurgitation TR; tricuspid regurgitation. *P* values in bold are statistically significant.

**Supplementary Table 2. Independent predictors of ΔPASP (pre- to 6-month post-TAVI).**

|  | Model 1 | | |  | Model 2 | | |
| --- | --- | --- | --- | --- | --- | --- | --- |
|  | Beta | 95% CI | *p* value |  | Beta | 95% CI | *p* value |
| Baseline Atrial fibrillation | -2.126 | -10.196 to 5.943 | 0.602 |  | -2.828 | -10.865 to 5.208 | 0.487 |
| Baseline 1◦ AVB | -2.269 | -12.243 to 7.705 | 0.653 |  | -1.670 | -11.607 to 8.267 | 0.739 |
| Baseline AR ≥ moderate | 6.723 | -0.675 to 14.121 | 0.074 |  | 5.893 | -1.599 to 13.386 | 0.122 |
| Baseline TR ≥ moderate | 5.933 | -1.492 to 13.357 | 0.116 |  | 6.297 | -1.449 to 14.044 | 0.110 |
| Moderate/severe paravalvular leak | -4.052 | -18.757 to 10.653 | 0.586 |  | -6.783 | -21.853 to 8.287 | 0.374 |

Model 1. Crude analysis; Model 2. Adjusted for BAV, Hypertension, Dyslipidemia, RBBB, AAD; 1◦ AVB, first-degree atrioventricular block; AR, aortic regurgitation TR; tricuspid regurgitation. *P* values in bold are statistically significant.

**Supplementary Table 3. Assessment of the proportional hazards assumption for Cox regression analysis of 1-year all-cause mortality.**

| Variable | χ^2^ | *p-*value |
| --- | --- | --- |
| Baseline AR ≥ moderate | 3.248 | 0.072 |
| Age | 0.106 | 0.745 |
| Baseline Atrial fibrillation | 2.801 | 0.094 |
| Baseline 1^◦^ AVB | 2.958 | 0.084 |
| Baseline CrCl | 0.006 | 0.940 |
| Baseline mPAD/BSA | 2.702 | 0.100 |

AR, aortic regurgitation; 1^◦^ AVB, first-degree atrioventricular block; CrCl, creatinine clearance; mPAD, main pulmonary artery diameter; BSA, body surface area; *P-*values in bold are statistically significant. *

**Supplementary Table 4. Assessment of the proportional hazards assumption for Cox regression analysis of 1-year cardiovascular mortality.**

| Variable | χ^2^ | *p-*value |
| --- | --- | --- |
| Baseline AR ≥ moderate | 3.047 | 0.081 |
| Age | 0.337 | 0.562 |
| Baseline Atrial fibrillation | 1.340 | 0.247 |
| Baseline 1^◦^ AVB | 2.333 | 0.127 |
| Baseline CrCl | 0.001 | 0.975 |
| Baseline mPAD/BSA | 1.866 | 0.172 |

AR, aortic regurgitation; 1^◦^ AVB, first-degree atrioventricular block; CrCl, creatinine clearance; mPAD, main pulmonary artery diameter; BSA, body surface area; *P-*values in bold are statistically significant. *

**Supplementary Figure**

**Supplementary Figure 1. Study flow chart.**


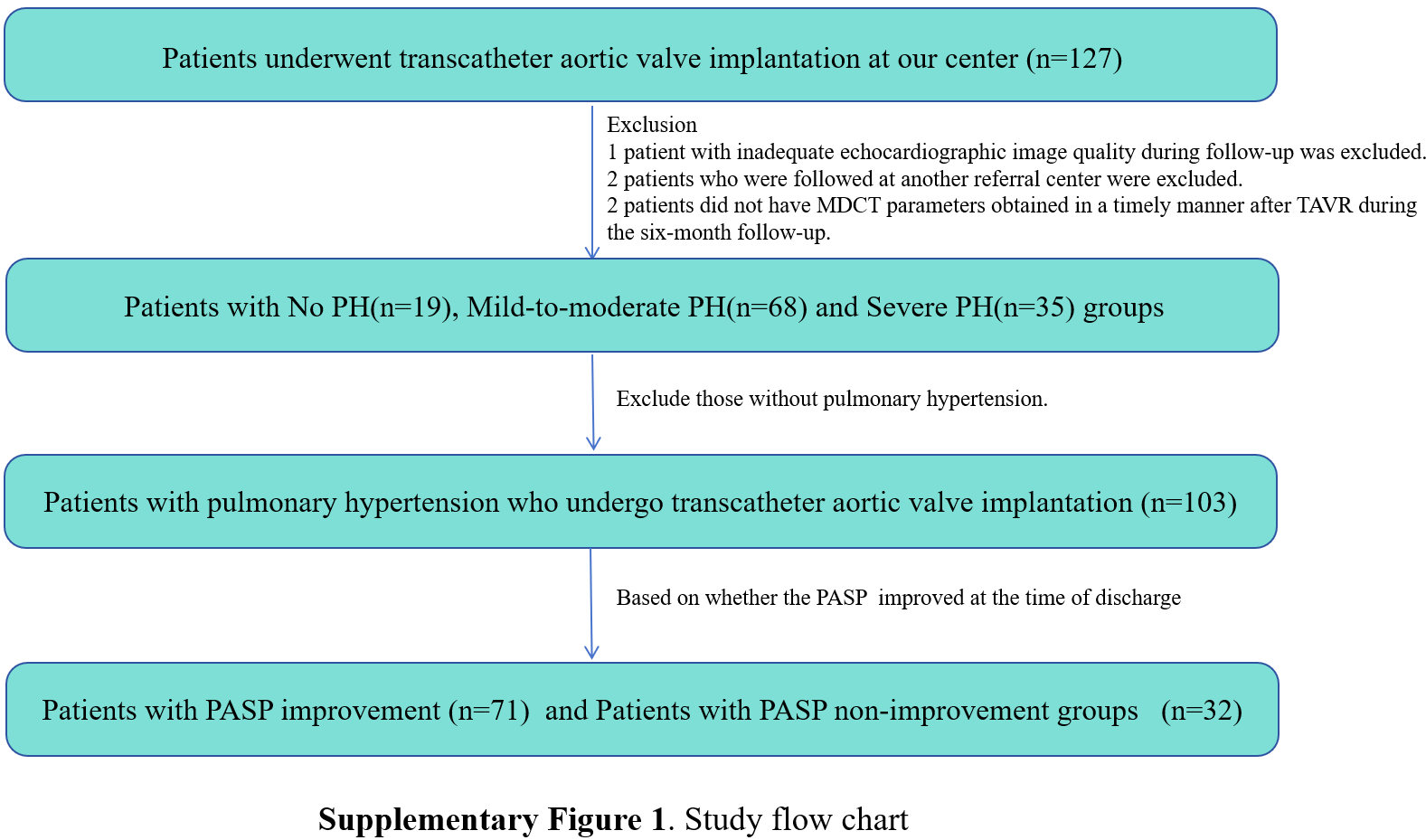

Supplement: Supplementary file 1 [file 2153-8174-27-6-50699-s1.zip › Supplementary Data.docx]
